# IoT-Based Framework for Digital Twins in the Industry 5.0 Era

**DOI:** 10.3390/s24020594

**Published:** 2024-01-17

**Authors:** Ahmed Awouda, Emiliano Traini, Giulia Bruno, Paolo Chiabert

**Affiliations:** Department of Management and Production Engineering, Politecnico di Torino, 10129 Turin, Italy; ahmed.awouda@polito.it (A.A.); emiliano.traini@polito.it (E.T.); paolo.chiabert@polito.it (P.C.)

**Keywords:** digital twin, Internet of Things, Industry 5.0, aeroponics, IoT-ARM

## Abstract

Digital twins are considered the next step in IoT-based cyber–physical systems; they allow for the real-time monitoring of assets, and they provide a comprehensive understanding of a system behavior, allowing for data-driven insights and informed choices. However, no comprehensive framework exists for the development of IoT-based digital twins. Moreover, the existing frameworks do not consider the aspects introduced by the Industry 5.0 paradigm, such as sustainability, human-centricity, and resilience. This paper proposes a framework based on the one defined as the outcome of a project funded by the European Union between 2010 and 2013 called the IoT Architectural Reference Model (IoT-A or IoT-ARM), with the aim of the development and implementation of a standard IoT framework that includes digital twins. This framework establishes and implements a standardized collection of architectural instruments for modeling IoT systems in the 5.0 era, serving as a benchmark for the design and implementation of an IoT architecture focused on digital twins and enabling the sustainability, resilience, and human-centricity of the information system. Furthermore, a proof of concept of a monitoring digital twin for a vertical farming system has been developed to test the validity of the framework, and a discussion of applications in the manufacturing and service sectors is presented.

## 1. Introduction

In recent years, after undergoing a transformative paradigm shift called Industry 4.0, the manufacturing landscape is now deeply immersed in the Fourth Industrial Revolution. This evolution involves the seamless integration of digital technologies, data analytics, and automation into conventional manufacturing processes, giving birth to what is commonly referred to as “smart factories”. Within these cutting-edge environments, cyber–physical systems (CPS), cloud computing, and the Internet of Things (IoT) collaboratively optimize efficiency, elevate productivity, and catalyze innovation [1]. At its core, the IoT interconnects devices and systems in a synergistic endeavor to augment industrial processes, extending its influence beyond typical computing devices [2]. This extensive network encompasses physical devices embedded with sensors and actuators, forming a real-time visibility network across the entire manufacturing ecosystem.

Digital twins (DTs) emerge as another pivotal element in this revolutionary wave. Serving as virtual replicas of physical objects or processes, digital twins facilitate data-driven decision-making, intricate systems monitoring, and product validation [3]. In the realm of cyber–physical systems, particularly in manufacturing, digital twins function as adaptive models that seamlessly integrate data between physical and virtual machines, holding transformative potential [4,5]. Digital twins, with the characteristics of ultra-high synchronization and fidelity, and convergence between physical and virtual products, have many potential applications in product design, product manufacturing, and product service [6]. One of the main benefits of digital twins is their ability to decouple physical flows from planning and control [7]; they create virtual replicas of physical assets and processes, allowing for simulation, real-time monitoring, and remote management. This decouples planning and control processes from physical flows by enabling efficient decision-making, optimization, and scenario planning in a virtual environment before implementation in the real world.

The symbiotic relationship between digital twins and the Internet of Things is evident in how IoT contributes data, and digital twins leverage these data to create dynamic, real-time models of their physical counterparts. This process involves integrating IoT data with virtual models, enabling the real-time monitoring, analysis, and optimization of physical entities [8].

Keeping well in mind the importance of designing information management systems that can work with human knowledge cognitive processes, in this paper, it is given as a hypothesis that such IoT systems should be able to manage industrial digital twin and complex knowledge management systems that are usually described as Industrial IoT (IIoT) systems [9].

Such complex Knowledge-Based Systems, well explained in [10,11], are considered to be based on hybrid modeling techniques, i.e., hybrid modeling is the process of making use of two or more modeling techniques belonging to different philosophies or methodologies and then synthesizing the results into a single score or spread. Hybrid modeling is conceptually similar to ensemble modeling but, while the second refers to the unification of different models of the same family, hybrid modeling refers to completely different approaches of modeling with a consequent increase in terms of the complexity of managing heterogeneous characteristics [12]. Moreover, hybrid knowledge in Industry 5.0 requires information systems with adequate information management platforms to digitize, manage, and use data involved in the information system: a more detailed description of the relationships between data, information, and knowledge is given in [11] through the DIKW method [13].

Despite the technological prowess of Industry 4.0 and its array of technologies, it is often perceived as a techno-economic vision. This vision outlines how broader technological advancements, originating outside industrial contexts, will influence industrial value chains and redefine the industry’s economic landscape [11]. However, Industry 4.0 falls short of effectively addressing the urgent environmental, social, and sustainability challenges of our contemporary world. Its emphasis on digitalization and AI-driven technologies for efficiency tends to overshadow the original principles of social fairness and sustainability. In response to these limitations, institutions and policymakers are shifting their focus toward human-centered design and ethical, responsible innovation in the factories of the future. This shift gives rise to the concept of Industry 5.0, introduced by the European Commission in 2020. Industry 5.0 aims to make production more sustainable, human-centric, and resilient, moving beyond shareholder value to embrace stakeholder value for all parties involved [14,15]. This vision is realized by acknowledging the capacity of the industry to attain societal objectives beyond mere employment and economic expansion. The aim is for the industry to transform into a robust source of prosperity by ensuring that production adheres to the limits of our planet and prioritizes the well-being of its workers throughout the production process. Furthermore, the focus is on utilizing technology not solely for economic gains, but rather for the advantage and convenience of all stakeholders involved [16].

The principles of Industry 5.0—human-centricity, sustainability, and resilience—build upon the technological foundations of Industry 4.0, placing the essential needs and interests of humans at the center of the production process [14,15,17]. This approach advocates for adapting technology to the needs of workers and ensuring that new technologies respect fundamental rights [18]. To achieve sustainability, Industry 5.0 promotes circular processes that rejuvenate, repurpose, and recycle natural resources, thereby reducing waste and environmental impacts [19]; this involves implementing innovative strategies and technologies. Rejuvenation refers to restoring resources through sustainable practices such as afforestation, soil restoration, and water conservation. Repurposing entails finding new applications or uses for materials, extending their lifecycle and reducing overall demand. Recycling involves the systematic collection and processing of materials to manufacture new products, minimizing the need for fresh raw materials. Resilience involves cultivating robust industrial production capable of withstanding disruptions and supporting critical infrastructure during crises [18].

Motivated by the glaring absence of IoT frameworks for digital twin development and the inadequacy of existing frameworks in meeting the requisites of Industry 5.0, this work proposes a framework rooted in the IoT Architectural Reference Model (IoT-A or IoT-ARM), i.e., a project funded by the European Union between 2010 and 2013. The proposed framework utilizes the IoT-A reference architecture for the base of structuring the IoT system as based on three sub-models: the domain, the information, and the functional model, to deal with the information systems managed by the IoT-A data and knowledge. Another main characteristic taken from the IoT-A reference is the formalization of the digital twin component acting in the system as a virtual entity. This concept is further elaborated in the following sections. Basing the proposed framework on the IoT-A one, there are many benefits like achieving interoperability, developing system roadmaps, managing product life cycles, and benchmarking in the IoT domain. This type of analysis is more deeply discussed by [20], from which the most important benefits described are briefly introduced below.

- Cognitive aid: IoT-ARM serves as a common language, facilitating discussions and communication among stakeholders involved in product development.
- As a reference model for common grounding, it establishes a shared understanding by defining IoT entities and their basic interactions, providing a common foundation for collaboration.
- The generation of architectures according to guidelines for translating the ARM into concrete designs.
- Identifying differences in derived architectures when using the IoT ARM, can be attributed to specific use cases and related design choices, providing insights into variations.

The proposed framework serves as a standardized collection of architectural instruments for modeling IoT systems, aligning seamlessly with the Industry 5.0 vision [21]. The significance of having a standardized set of architectural instruments ensures interoperability, compatibility, and scalability across diverse IoT ecosystems, fostering seamless integration and communication among heterogeneous devices and platforms. This standardization not only streamlines the development process but also facilitates data exchange, enhancing the reliability and efficiency of digital twin implementations. Additionally, it promotes a common language and framework, enabling cross-domain collaboration and accelerating innovation in the rapidly evolving landscape of IoT-based digital twins. Ultimately, a standardized collection of architectural elements serves as the linchpin for achieving cohesive, reliable, and scalable IoT architectures that underpin the robust development and deployment of digital twins across various domains [22].

Unfolding in subsequent sections, this article elucidates the state of the art and delineates the methodology employed for the literature review in Section 2. Section 3 delves into the intricacies of the developed framework, while Section 4 scrutinizes the case study in which the framework has been applied. Finally, Section 5 encapsulates the primary findings and conclusions drawn from this endeavor.

## 2. State of the Art

The literature review begins with the outcomes of the European Lighthouse Integrated Project, IoT-A, that aimed to address the IoT interoperability and scalability challenges alongside challenges faced when modeling IoT systems. These outcomes are summarized in the book “Enabling Things to Talk: Designing IoT solutions with the IoT Architectural Reference Model” [20].

**Figure 1:**
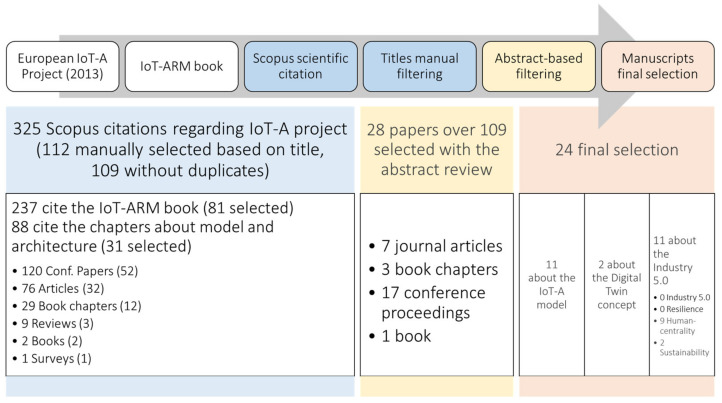
Summary of the literature review process and results from the forward citations research: manuscripts citing IoT-A on Scopus. European IoT-A Project (2013) [23].

The first step of the literature review method was a forward citation process that was conducted on Scopus to find all the articles that cited IoT-A to understand how the outcomes of this project contributed to the scholarly conversation. The main components of this process are presented below, and the review methodology is highlighted in Figure 1.

- Initially, 237 documents were found to have cited the book and 88 more to have cited specific chapters of it, leading to 325 documents.
- After that, manual filtering based on the article titles was done to include relevant articles dealing with IoT-A and to exclude any that were far from topic, resulting in 109 documents without duplication. These documents are composed of 32 journal articles, 52 conference papers, 3 review articles, 12 book chapters, 2 books, and 1 survey paper.
- The following step in the literature review process was abstract-based filtering. This involved going through all the abstracts and extracting articles that have a specific focus or contribution to IoT-ARM. Moreover, since the objective of this work is to provide a framework enabling digital twins that takes into consideration social and human aspects addressed by Industry 5.0, another extraction criterion was included in the abstract search. Specifically, any articles touching on digital twins, Industry 5.0, sustainability, human-centricity, and resilience were extracted. This process resulted in 28 articles that fit the extraction criteria. These contain 7 journal articles, 3 book chapters, 17 conference papers, and 1 book.
- The final step was a deep review of these documents focusing on contributions to IoT-ARM and keywords related to Industry 5.0 and the digital twin concept. It was found that 5 documents mention the digital twin concept while only 2 of them provide meaningful contributions inherent to the scope. More than this, 11 articles introduce and delve into IoT-ARM with only 3 of them providing considerable contributions. And finally, 11 documents touch on concepts related to Industry 5.0; however, none of them explicitly mention Industry 5.0, but only related concepts such as sustainability (2) and human-centricity. Direct contributions to resilience were not found. The analyzed manuscripts, the ones that are the results of the research method described in the figure, fall within three main themes: comparison between various IoT architecture reference models, discussion of applications in specific domains, and detailed contributions to the IoT-A Framework.

The authors of [24] compared various IoT reference architectures to the IoT-ARM, with a focus on reference architectures that enable IoT integration with cloud computing or fog and edge computing; they also highlighted the importance of socio-technical perspectives when dealing with IoT and highlighted the importance of associate actors and processes which IoT interacts with. Ref. [25] also compared IoT reference architectures using a quantitative approach; they provided a set of criteria that can be modified based on the requirements of the application and the end users. Ref. [26] proposed a strategy to integrate the methods and architecture of IoT with cyber–physical systems and multi-agent systems. The authors of [27] demonstrated the application of IoT-ARM in generating system architectures for IoT platforms in the smart city sector with a focus on security, privacy, and scalability. The developed platform provides architectural artifacts for efficient and scalable security and user-centric privacy.

Ref. [28] introduced a conceptual framework for designing and implementing digital twins, tailored towards the farming sector; their framework consists of a control model based on a general systems approach and an implementation model based on the functional model of IoT-A. The work of [29] also proposed a reference architecture based on IoT-A for the development and implementation of digital twins in the agricultural sector. They built on the work of [28] by defining a set of architectural views that address different stakeholder concerns. More specifically, they focused on the functional decomposition and deployment views of IoT-A but tailored these towards digital twin development. The authors of [30] introduced different model-based approaches used in the lifecycle of IoT application development. They highlighted the benefit of IoT-ARM in the fact that it not only deals with the physical layer of an IoT system but also on the digital twin and the application-specific components.

The work conducted by [31] proposed a new IoT communication reference model based on the IoT-A communication model. According to them, the original communication model does not suitably handle the security and quality of service features; to this end, they introduced a security and a quality-of-service (QoS) layer. Ref. [32] proposed a framework that encompasses a model-driven approach for applying QoS attributes in the development of the IoT systems. They emphasize the importance of introducing quality attributes such as confidentiality, scalability, reliability, and fault tolerance into the IoT-A modeling process. Another contribution of their work is the extension of the domain and information models of IoT-ARM by adding a perspective metamodel and linking it to each of them.

The usage of IoT-A to achieve Industry 5.0 objectives is not highly emphasized in scientific literature; out of all the literature that cites IoT-A, there is a very limited number of contributions to sustainability, human-centricity, and resilience. In [33], the authors underscored the pivotal role of the IoT in fostering a sustainable world and enhancing human well-being. Their work delves into both hardware and software approaches aimed at minimizing energy consumption in IoT-based services. By providing insights into strategies deployed at different levels of IoT-based services, the authors contribute to the overarching goal of reducing power consumption and fostering a more sustainable environment. The comprehensive approach presented in their work aligns with the imperative of creating IoT solutions that not only advance technological innovation but also prioritize energy efficiency for long-term environmental and human well-being benefits.

With regard to human inclusion in IoT reference architectures, Ref. [34] introduced a human–computer interaction quality evaluation method based on ubiquitous computing software measures, while employing IoT-ARM as the reference architecture for their implementation. More specifically, they introduced the human actor as part of the IoT-A domain model and provided insight on how to link the human model to the machine and platform model considering them as multiple agents. They also highlighted the importance of providing the human worker with context-based information reduction to minimize information overload. The paper written by [35] presented an architecture based on IoT-A and subject-oriented process representations which allows one to encode and adapt to human properties on different levels of an organizational control process; they highlighted the importance of continuously adapting manufacturing system behavior to worker needs by incorporating human-aware modeling. Refs. [36,37] also highlighted this aspect by emphasizing the vital role of Humans in Production Industries and the need for modeling human interactions in such systems. The concept of humanized IoT was introduced by [38] by applying Fiskes’s Four Elementary Forms of sociality to IoT, they developed a personal and communal IoT platform that allows users, with different profiles and skills, to share information, interact and build on top of IoT applications. Considering the role of humans in cyber physical systems a hierarchy of design level for socio-technical systems was introduced by [39] and was developed according to the IoT-A domain model.

One of the more recent works was a review conducted on the role of human digital twins (HDTs) in the context of Industry 5.0 [40]. The authors conducted a comprehensive survey and proposed a conceptual framework and architecture for HDTs that emphasizes interactions, responsibilities, and collaborations between components. They also highlighted the importance of IoT as an enabler for human DTs due to its ability to integrate all the actors in the manufacturing ecosystem. Another review carried out by [41] analyzed the application of digital twins in the context of Industry 5.0. Their review found that DTs can generate value, shorten the time to market, enhance plant machinery and final product performance, and offer unique insight not found in any other solution.

## 3. IoT-DT Framework

### 3.1. Overview and First Considerations

The proposed framework aims for a 5.0 characterization by deciding the structure of the information model, functional model, and information view in alignment with the IoT-A reference architecture. Additionally, a key focus is placed on designing the IoT system around digital twins, emphasizing the connection between physical entities and their corresponding virtual representations. To achieve these goals, specific requirements are addressed in the subsequent section, aligning with the contextual demands of the 5.0 framework.

**Figure 2:**
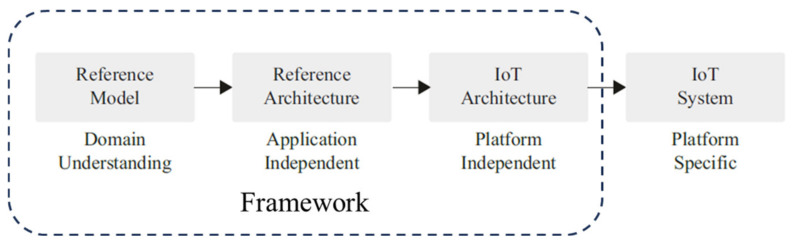
Framework application in IoT development process, adopted from [24].

The proposed approach starts by defining the generic requirements of industrial IoT systems [42], then, these are complemented by Industry 5.0 requirements that focus on human-centricity, sustainability, and resilience that form the basis for the KPIs developed. Furthermore, a methodology for implementing these requirements into an IoT-a-based domain model is defined [43], along with the sub-models that complement it, namely an information, functional, and communication model. Based on this model and frameworks found in the literature, a system architecture is proposed. Figure 2 shows the general steps followed throughout the framework and the derivations of every step.

**Figure 3:**
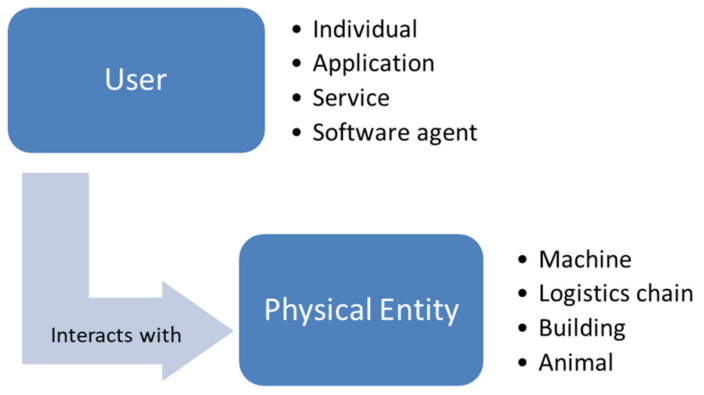
Basic IoT interaction as defined by [20].

In accordance with the IoT Architectural Reference Model (IoT-ARM) [20], the prototypical IoT scenario delineates a generic user interfacing with a physical entity (PE), which may be spatially distant, within the tangible domain, as depicted in Figure 3. In the physical realm, direct interactions, exemplified by the manual relocation of an assembled part from point X to Y, are operationally viable. Nevertheless, the core tenet of IoT lies in enabling indirect engagements through a third-party intermediary. This is manifested through the invocation of a Service capable of imparting information about the physical entity or effectuating a specified action upon it. Thus, the framework is categorically denoted as service-oriented, underscoring this inherent dynamism. In practical instantiation, a human user gains entry to a service through a service client—a software entity featuring an accessible user interface. This access modality aligns seamlessly with the service-oriented paradigm of the framework, accentuating the capacity to interact with Physical Entities through services facilitated by software interfaces. The limitations of interacting with physical entities through services facilitated by software interfaces may arise from various technological, practical, and ethical factors. The main limitations are the hardware dependencies, since interacting with physical entities often requires compatible hardware components, and connectivity issues, since poor or unstable connections can lead to communication failures or delays, impacting the effectiveness of remote control and monitoring. Other limitations can be the latency and response time, the security concerns, the privacy of the data, and the energy and resource constraints. It is possible to address these limitations by using a combination of technology advancements, robust security measures, and thoughtful design, to ensure safe and effective interactions with physical entities through software interfaces. The orchestration of such interactions embodies the intrinsic technicality and sophistication integral to the engineering and scientific underpinnings of this IoT framework.

It is noteworthy to mention that the main component of the IoT-A models is the virtual entity; this is intuitive and in line with the notion of “things” in IoT since physical entities are the “things” and virtual entities are representations of these physical entities in the information system. However, this definition of a virtual entity is tightly coupled with other concepts in the IoT realm such as digital twins, digital shadows, and digital models. According to [44], a digital twin is a collection of adaptive models designed to mimic the behavior of a physical system within a virtual environment, continuously updating in real-time throughout the system’s life cycle. This digital twin replicates the intricacies of the physical system, enabling the anticipation of potential failures and identification of opportunities for change. Through this emulation, the digital twin can prescribe real-time actions, optimizing and/or mitigating unexpected events by keenly observing and evaluating the operational profile of the system. The essence lies in the synchronized, dynamic relationship between the physical and virtual representations, fostering proactive decision-making and enhancing the overall system performance.

**Figure 4:**
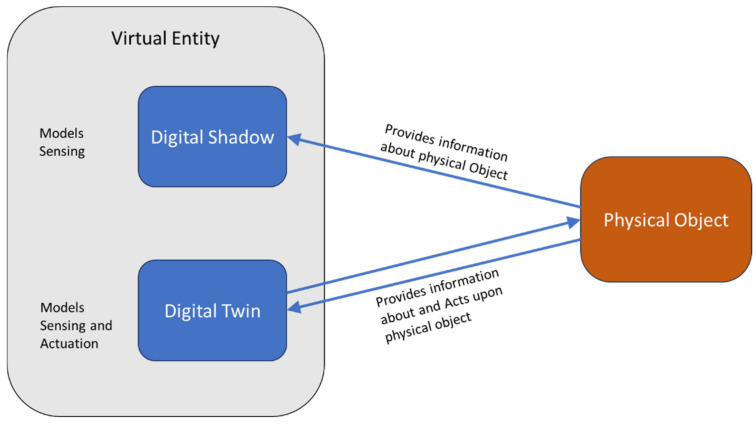
Virtual entities in relation to digital twins and digital shadows.

Considering this definition, a DT is a virtual entity with specific characteristics and highly complex interactions. On the other hand, a digital shadow is a virtual model that represents the physical model only, with one-way data flow [45]. A digital shadow is characterized by a unidirectional data flow, meaning data can only go from one location to the other. It is possible to make digital shadows of digital twins because they can capture and simplify the multitude of information that passes through a twin; however, a digital shadow cannot influence its physical counterpart or conduct an action upon it (actuation), unlike the digital twin. We highlight the difference between these terms in Figure 4. IoT-A goes further and defines the augmented entity as a combination of a virtual entity and its physical counterpart. However, we have omitted this entity as it contains no additional information about the physical entity than ones contained in the virtual entity, so it does not really provide any additional functionalities found in a digital twin. Furthermore, since the concept of digital twins was fairly unknown when IoT-A was developed, we argue that the augmented entity was an attempt to model digital twins in IoT systems and since virtual entities can fit this description of DTs, we felt no added value was provided by including this entity in the domain model.

To capture the various complexities of an IoT system, a single model will not suffice; on the contrary, multiple models that capture different aspects of the system are required. In the present work, three models are considered, namely the domain, information, and functional. The virtual entity can be considered the interaction point for these models, since the domain model considers all elements of the IoT domain. The information model can be considered a breakdown of the virtual entity since all the information about the “thing” is contained in the virtual entity. Thirdly, the functional model identifies groups of functionalities that build on each other following the relations identified in the domain model. The functionalities of these functional groups that manage information use the IoT information model as the basis for structuring their information, and therefore are closely linked to the virtual entity concept. These models will be explained in more detail throughout this section.

It is noteworthy to mention that IoT-A includes a security, trust, and privacy model that interacts with the functional model at the security layer (as shown in Section 3.5). This model focuses on trust at the application level, by defining a trust mechanism that provides data integrity and confidentiality, and endpoint authentication between any two system entities that interact with each other. Moreover, it defines a security reference model that includes application security, service security, and communication security. Furthermore, it includes a privacy sub-model that describes the privacy measures that stop a subject’s (either user’s or entity’s) data from being misused, such as access controls, encryption/decryption methods, and credential-based security measures.

While security is a very important aspect and has high implications for the success and failure of IoT deployments [46], it is beyond the scope of this article to delve into the intricacies of this deep discussion; however, Ref. [23] provides a broader discussion about the security, trust, and privacy model.

### 3.2. Industry 5.0 Requirements

System requirements are usually categorized in terms of functional and non-functional requirements. Functional requirements outline what a system should do by specifying its features and capabilities from a user’s perspective, while non-functional requirements are not directly observable by end-users. While functional requirements focus on the users and system interaction and they are closer to the user interface (the ability for users to transfer funds between accounts, for instance, in a banking system), non-functional requirements define how the system should perform, emphasizing qualities like performance, security, reliability, and scalability. When talking about IoT systems specifically, several general functional requirements are usually defined for the developed system, which can include real-time data acquisition from sensors, the reliable transmission of data, and device management, to name a few. These requirements are implemented as features by the developer. Several features may need to be present to implement a single requirement, and vice versa, a single feature can satisfy multiple functional requirements.

The emphasis of this work is not on supplementing functional requirements for IoT systems; instead, the focus lies on non-functional requirements, specifically those essential for Industry 5.0 compliance. Considering non-functional requirements as expressions of how functional requirements are achieved, the goal is to formalize the analysis and management of how the IoT system should operate during development, ensuring alignment with functional requirements. Given the core tenets of Industry 5.0—sustainability, human-centricity, and resilience—the formalization of I5.0 requirements must inherently incorporate these pillars. The central discussion revolves around translating the key attributes of human-centricity, resilience, and sustainability into system requirements. The identified requirements, derived from the Industry 5.0 vision, are intended to be integrated into the framework. The framework is expected not only to facilitate an assessment of system conformity to these requirements but also to serve as the foundation for developing indicators and metrics for evaluating Industry 5.0 conformity.

#### 3.2.1. Human-Centricity

Human-centricity in IoT systems refers to the prioritization of human needs, motivations, and beliefs in the design and implementation of these systems [47]. This approach is usually applied in the context of Internet of Medical Things (IoMT) systems, where it can lead to improved user satisfaction and system functionality [48]; however, it should not be limited to this sector but rather to all IoT systems that provide services to humans or have any form of human interaction.

Human-centricity requirements synthesized from [49] pivot on safeguarding human potential and prioritizing humanity over the exploitation of vulnerabilities. These requirements, rooted in a commitment to placing human welfare at the forefront, can be summarized as follows:

- Emphasize the role of both the designer and the human user in shaping the overall experience with the result of active human involvement, where the synergy between human creativity and technological design contributes to a richer experiential landscape;
- Consider the human user as belonging to a society, and this society, restricted to the corporate system and not, builds the experience and thus the wisdom that constitutes the know-how of humans at the basis of a system, in this case, of a production system;
- Promote an environment that empowers individuals to exercise their self-direction and creativity (not individualism, but the enhancement of the individual);
- Value people beyond their role as “users,” recognizing the needs and contributions of the individual, transcending conventional labels and standardization to ensure a holistic and respectful approach to human interactions with technology.

#### 3.2.2. Sustainability

In terms of sustainability, the Brundtland report [50] introduced the principles of environmental, social, and economic sustainability as the three dimensions of sustainability and sustainable development. The sustainability of IoT systems is a critical concern, given their potential to contribute to energy consumption, toxic pollution, and electrical waste. The proliferation of IoT devices contributes to increased energy consumption, often relying on non-renewable sources, leading to a higher carbon footprint. Additionally, the manufacturing, use, and disposal of these devices can result in toxic pollution due to the presence of hazardous materials. The rapid pace of technological advancements and device obsolescence further contributes to electrical waste, exacerbating the global e-waste problem. Several studies have explored the concept of “green IoT” to address these challenges. Refs. [51,52] both emphasize the importance of green sensing and communication in IoT systems, with the latter proposing a framework for minimizing carbon footprints and promoting the use of green IoT.

- Sustainability, defined in [53], is a concept based on three main aspects:
- The environmental aspect, with environmental KPIs (e.g., carbon footprint, energy consumed per message);
- The economic sustainability, with the indicator for the cost of information (e.g., cost of sending a message);
- The social impact, especially on users outside the system boundaries.

#### 3.2.3. Resilience

Resilience in IoT systems refers to their ability to withstand and recover from malicious activities, disruptions, and failures. This is particularly important given the complex and dynamic nature of these systems [54,55]. The concept of resilience is crucial in the design and operation of IoT systems; however, the practical implementation of resilience mechanisms in IoT systems can be challenging, as they impose constraints on developers [56]. Despite these challenges, the need for resilient IoT systems is clear, given their increasing integration into critical infrastructures. An example of a resilient IoT system is a smart energy grid that incorporates self-repairing capabilities [57]. Through the real-time monitoring and analysis of grid performance, the system can quickly identify faults or disruptions. Autonomous responses, such as rerouting energy flows or isolating affected areas, ensure the continuous and reliable supply of electricity. This resilient IoT application enhances the stability of the energy grid, minimizing the impact of failures and contributing to the overall resilience of the critical infrastructure. Other examples include intelligent transportation networks with adaptive traffic management that can self-adapt to mitigate congestions [58]. Other examples in smart manufacturing facilities include systems with predictive maintenance strategies, forecasting potential equipment failures and optimizing operational efficiency [11,59,60]. Additionally, these systems excel in fault diagnosis by promptly identifying and diagnosing anomalies in real-time data streams [61,62,63].

- Resilience, i.e., being future-proof and able to adapt to disturbances is considered as the following:
- The ability to adapt to disturbances;
- A sufficient scalability level;
- The estimated quality level of the data sent.

### 3.3. Domain Model

The IoT domain model defines the fundamental components of the IoT. Additionally, it describes the fundamental features of these concepts, such as the name and identifier, as well as the relationships between concepts [64]. The primary objective of a domain model is to develop a shared knowledge of the target domain and to capture the key concepts and relationships that are pertinent to IoT stakeholders. The domain model defines various types of elements that make up its basic building blocks and allow for the modeling of various characteristics and systems within an IoT environment.

The first is the physical entity, an observable component of the physical world relevant to the user’s objective. Physical entities may be practically any object or environment, including persons, animals, automobiles, retail or supply chain products, computers, electrical equipment, jewelry, and clothing.

Virtual entities are representations of physical entities in the digital world. There are many kinds of digital representations of physical entities: 3D models, database entries, objects (or instances of a class in an object-oriented programming language). Active or passive classifications can be applied to virtual entities, Active Digital Artifacts (ADA) are software programs, agents, or services that have access to other services or resources. Passive Digital Artifacts (PDA) are inert software components, such as database records, that can serve as digital representations of physical entities. For an element to be considered a virtual entity it must satisfy two fundamental properties:

- Virtual entities are coupled with a single physical entity, which the virtual entity itself represents; however, a single PE can be represented by multiple unique VEs, providing a distinct representation for each application domain;
- Synchronization, i.e., VEs must provide synchronized representations of a specified set of features (or qualities) of the physical entity, i.e., a change in the physical world must be reflected in the physical entity and vice versa.

Devices, as technological artifacts, serve as interfaces bridging the gap between the digital and physical realms, establishing a connection between virtual entities and physical entities. Consequently, these devices must exhibit functionality in both the physical and digital domains. However, the IoT domain model predominantly focuses on its capability to facilitate observation and the modification of the physical environment from the digital sphere. If other attributes of a device are significant, the device itself would be modeled as an entity. In line with the IoT-ARM specifications, three primary types of devices are defined: sensors, actuators, and tags.

Resources, within the IoT context, refer to software components that either provide data sourced from physical entities or are integral to their operational processes. On-Device Resources are discernible from Network Resources. As the name implies, On-Device Resources reside locally on devices, comprising software installed directly on the device connected to the physical entity. These resources encompass executable code designed for accessing, analyzing, and storing sensor data, as well as code for controlling actuators. In contrast, Network Resources refer to resources accessible over the network, such as cloud-based databases. This distinction underscores the essential role that both On-Device and Network Resources play in the overall architecture of the IoT ecosystem.

Services, as defined by [65], serve as the mechanism for aligning requirements with capabilities. Within the IoT framework, services are confined to technical services delivered through software. They function as the interface linking the IoT components of a system with other, non-IoT-specific elements within an information system, such as enterprise systems. The orchestration of both IoT-related services and non-IoT services allows for the comprehensive construction of a system. Unlike heterogeneous resources, which may heavily depend on the underlying hardware of the device for their implementation, a service provides an open and standardized interface encompassing all the essential functionalities required for managing resources and devices associated with Physical Entities. Within the service hierarchy, low-level services play a pivotal role by directly interacting with resources and residing closest to the actual hardware of the device. These low-level services may be further invoked by other services to deliver higher-level functionalities, such as the execution of a business process activity. This hierarchical structure highlights the layered nature of services within the IoT spectrum, where low-level services form the foundation, facilitating interaction with device hardware, and higher-level services build upon this foundation to enable complex functionalities and business processes.

**Figure 5:**
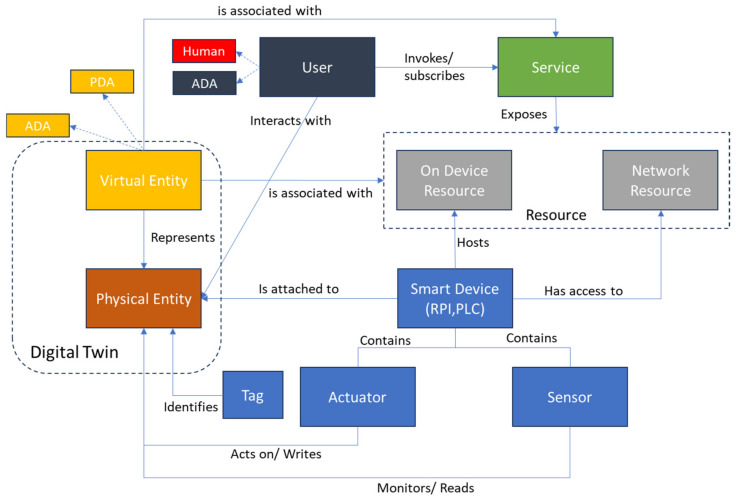
IoT domain model.

Figure 5 shows the IoT domain model which highlights the relationships between the above-mentioned elements. The model shows a physical entity that has a device attached to it; we use the term smart device (in contrast to the original device term used by IoT-A), basing our definition on [66], to indicate an electronic component that is able to connect, share, and interact with its user and other smart devices; furthermore, this definition of a device can encompass sub-device classes such as sensors that can directly interact with IoT systems without the need for an interface (such as a PLC or a gateway), which is the case in many Industry 4.0 environments found today. The smart device can contain a sensor or an actuator that monitors or acts on the physical entity. The smart device hosts on device resources and can utilize Network Resources. These resources are exposed to the user via a service that the user can invoke or subscribe to. The physical entity is represented in the digital realm using a virtual entity, which in certain cases, becomes the digital twin entity. The virtual entity is associated with a specific set of resources that are exposed to it via a service. The relationship between virtual entities, resources, and services will be elaborated more in the information model section.

The significance of the domain model stems from its incorporation of fundamental abstract concepts, outlining their roles and interconnections. Concerning granularity, the domain model should distinguish between relatively constant elements and those subject to variation. For instance, in the IoT domain, the concept of a device is likely to endure, even as the specific types of devices evolve over time or differ based on the application context. As a result, the model refrains from detailing specific technologies, instead focusing on abstract representations.

Moreover, due to the abstract nature of the domain model, its applicability in various industries becomes part of the core nature of the framework. In the current work, the framework is applied to vertical farming, but it can be expanded beyond this sector to other sectors beyond it such as manufacturing and service management. Furthermore, the core elements of an IoT system are shared among the various sectors, allowing for cross-sector applications of the technology. These elements include perception for data collection and interfacing the physical and digital worlds, networks and communication for connecting the IoT ecosystem, middleware (platform layer) for data processing and analytics, and finally, an application layer for decision support and integration with business systems.

### 3.4. Information Model

On a conceptual level, the IoT information model describes the structure (e.g., relationships, characteristics, and services) of all the information for virtual entities. The virtual entity is the key concept of any IoT system, as it models the physical entity or the thing that is the real element of interest. The word information is used in conjunction with the definitions of the DIKW hierarchy [11,67], where data are defined as values devoid of context that is meaningful or useful. Information provides context for data and answers to common queries such as who, what, and when.

**Figure 6:**
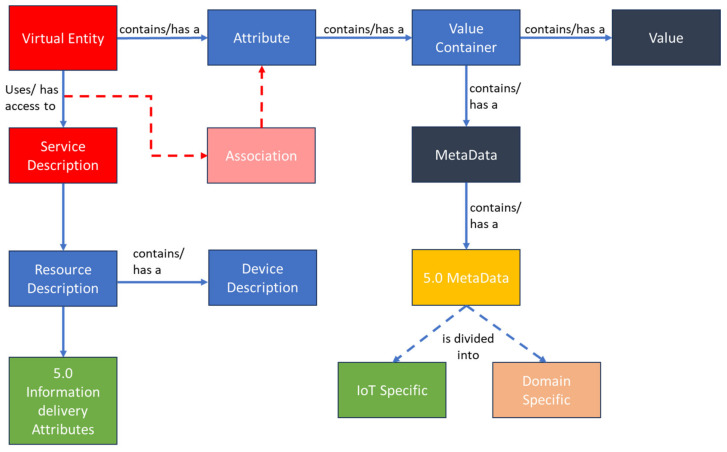
IoT information model, adopted from [20].

The IoT information model provides information on the modeling of a virtual entity. As shown in Figure 6, the virtual entity (virtual entity) contains attributes with a name, a type, and one or more values that can relate to metadata (MetaData). Metainformation includes, for instance, the unit of measurement, the time at which and the location where a value is digitized, or the quality of this digitization process, i.e., the quality, for example, in terms of the veracity of the measure. The virtual entity is interfaced to the remainder of the system (sources of information) by its ability to access a service through the description of the service itself that describes how a service serves information.

Our contribution to the IoT-A information model is the modification of the virtual entity to make it compliant with Industry 5.0. This is done by formalizing a description of how the service, resource, and device descriptions handle information related to Industry 5.0, and by defining a structure for the MetaData component that will contain the metainformation pertaining to the Industry 5.0 requirements described previously.

The association between a virtual entity and a service is specified by a particular attribute of the virtual entity. The type of the service can be decided to be either information or actuation, depending on whether it offers value to be read or written.

A service description describes the relevant aspects of a service, including its interface. Additionally, it may contain one (or more) resource description(s) describing a resource whose functionality is exposed by the service. The resource description in turn may contain information about the device on which the resource is hosted.

With regard to the Industry 5.0 information delivered to the virtual entity, it is eventually all encompassed in the service description in some form of container; when linking these attributes to the virtual entity, the same method of association described above is used, i.e., through an association, the connection between an attribute of a virtual entity and the service description is modeled. However, once they are part of the virtual entity, they will not be modeled as one or more attributes but as MetaData, since they fit the definition of metadata or meta information as being “information about the value of a piece of information” [68], to this end, the block Industry 5.0 metadata are added.

To summarize, the IoT information model is a metamodel that provides a structure for the information being handled by IoT systems. This structure provides the basis for all aspects of the system that deal with the representation, gathering, processing, storage, and retrieval of information, and as such, is used as a basis for defining the functional interfaces of the IoT system.

### 3.5. Functional Model

Functional Decomposition (FD) refers to the process by which the different Functional Components (FCs) that make up the IoT ARM are identified and related to one another [69]. The primary objective of Functional Decomposition is, on the one hand, to reduce the complexity of an IoT ARM-compliant system into smaller, more manageable components, and, on the other hand, to comprehend and show their relationships. IoT-A defines the functional model as “an abstract framework for understanding the main Functionality Groups (FG) and their interactions”. This framework defines the common semantics of the main functionalities and will be used for the development of IoT-A-compliant Functional Views.

The IoT functional model used in this framework is adopted from [28], which is based on the original IoT-A functional model and its modifications suggested by the same authors in [70]. The main addition provided by this “improved functional model” is that the virtual entity management is replaced with digital twin management.

**Figure 7:**
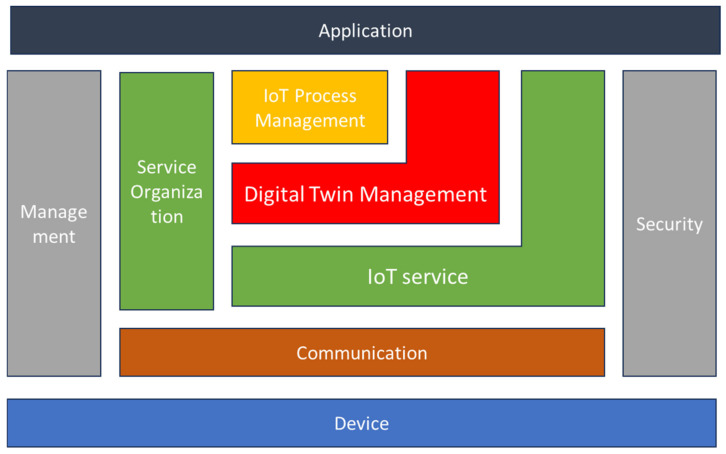
IoT functional model, adopted from [28].

As shown in Figure 7, the functional model addresses eight layers (functional groups), ranging from a device layer, which is attached to the physical entities, to an application layer, which allows various user interactions. The device layer provides the hardware components that are attached to and directly interact with physical objects as defined by the IoT domain model entity of device; this includes sensors and actuators.

The communication layer regulates the relationships between the various components and facilitates communication from the devices to the IoT services. It facilitates end-to-end communication across diverse networking contexts by providing networking, connection, and data transport capabilities. Moreover, it abstracts the heterogeneous interaction schemes stemming from the myriad technologies associated with IoT systems, encapsulated within the device Function Group (device FG), and establishes a unified interface to the IoT Service FG. This abstraction serves to simplify the instantiation and management of high-level information flow. Specifically, it addresses various aspects of the ISO/OSI communication model, encompassing considerations related to data representation, end-to-end path information, and network management. This unified interface ensures a cohesive and standardized approach to handling communication intricacies within the IoT ecosystem, providing a streamlined mechanism for the efficient instantiation and management of information flows at a higher level of abstraction [71].

The IoT service layer includes services and features for IoT service discovery, lookup, and name resolution. It can be used to retrieve data from a sensor device or send data to control actuator devices. As mentioned previously, sensors and actuators capture and facilitate the change in certain aspects of the physical world. The resources associated with the sensors and actuators are exposed as IoT services on the IoT service layer. Examples of applications and IoT system interactions in this layer can be “Give me the value of Sensor 123-55” or “Set Actuator 123-31 to On”.

The digital twin Management layer contains functions for interacting with the IoT system based on virtual entities. It can give access to all the information about the digital twin, from sensor devices, databases, or applications. Furthermore, it contains all the functionality needed for managing associations with physical objects and monitoring their validity. The relationship between the virtual entity FG and the IoT service layer is close one, this is because this layer models higher-level aspects: examples are “Give me the outdoor temperature of Car 123” or “Set lock of Car 123 to locked”.

The IoT Process Management FG is concerned with the integration of process management activities given by the business with the IoT system. Its overarching objective is to furnish the functional concepts requisite for seamlessly integrating the peculiarities of the IoT realm into conventional (business) processes. This layer establishes an environment conducive to the modeling and execution of processes that are cognizant of IoT considerations. The deployment of process models to execution environments is facilitated through the utilization of IoT Services. It is crucial to note that this layer is inherently proximate to enterprise systems. Therefore, the modeling of processes within this context must not only account for the nuances of the IoT domain but also incorporate the specificities of the underlying business domain. This dual consideration ensures a comprehensive and coherent approach to process management, acknowledging both the intricacies of IoT functionalities and the broader context of enterprise operations.

The Security layer is responsible for the security and privacy of the systems and its users. It handles the initial registration of a client to the system in a secure manner and protects the private data of users. It also ensures legitimate interactions between peers that are authorized to interact with each other, and it manages secure communications.

The service organization layer acts as a central hub between the other functional groups; this is important since the final objective is to achieve a service-oriented model or architecture. The IoT Process Management FG relies on Service Organization to map the abstract process definitions to more concrete service invocations. Moreover, it enables the translation of high-level requests to specific IoT services, therefore also linking the digital twin management to the IoT service layer.

Finally, the Management layer indeed plays a crucial role in overseeing and controlling the following aspects of the system according Industry 5.0 guidelines: system setup and configuration (for example, adding devices and communication protocols), error reporting (monitoring activity for maintaining system reliability and performance, i.e., to ensure resilience), system health monitoring (sustainability real-time assessment), strategic decision supporting and functionalities execution management (defining and implementing strategies to guarantee that the functions and actions of the resulting IoT system contribute to achieve the 5.0 goals in terms of human-centricity, sustainability, and resilience.

The human-centricity aspect of the Management layer, for example, is guaranteed by a management system of all the functions given by the proposed functional model (summarized by Figure 7 between the applications and devices of the IoT system) in order to pursue measurable goals thanks to several human-centric functions of the whole information system. The following are examples of functions, or groups of functions (services), to be made sustainable through the design of an IoT system to support the management of the information system in which users (humans) and resources operate.

- Flexible work arrangements, allowing for smart (flexible and remote) working hours and a personal level of responsibility for processes.
- Encourage open communication between employees to share their ideas, concerns, and feedback.
- Provide learning opportunities to support continuous learning and to encourage employees to pursue projects or initiatives that align with their personal and professional development goals.
- Recognition and rewards for creativity, implementing a recognition system that rewards employees for innovative ideas and creative problem-solving (impossible to consider during the IoT design).
- Diversity and inclusion towards a diverse and inclusive environment for humans from various backgrounds (without digital barriers of IT platforms).
- Supportive leadership by cultivating all human leadership styles, following clear guidance, and acting as a facilitator rather than an authority.
- Experimentation and risk-taking, promoting the culture of learning from failures, creating a safe space.
- Provide tools and resources (access to tools and technologies that are needed to pursue human ideas and projects).

## 4. Case Study

### 4.1. Overview

The global population is projected to reach an estimated 8.5 billion by 2030 and is anticipated to further increase to 9.7 billion by 2050 [72]. This substantial population growth necessitates a significant boost in agricultural production to ensure food security. However, the expansion of such production is constrained by environmental crises and the adverse effects of conventional open-field agricultural practices [73,74]. To overcome these challenges, smart farming techniques, such as aeroponics, offer a promising avenue for optimizing resource utilization. In accordance with [28], a smart farming system can be conceptualized as a cyber–physical control cycle, seamlessly integrating sensing and monitoring, smart analyses and planning, and the intelligent control of farm operations across all pertinent processes. However, the successful implementation of these modern agricultural techniques relies heavily on reliable and up-to-date information about farm operations. This is precisely where digital twins (DTs) come into play, providing a valuable tool to enhance and optimize agricultural practices by offering real-time insights and data for informed decision-making. In this context, DTs can help farmers get information on the fields and overall farm behavior, and thus decide better crop management strategies. By collecting data, it can also forecast yield prediction, growth stage, nutrient information, and weather reports.

**Figure 8:**
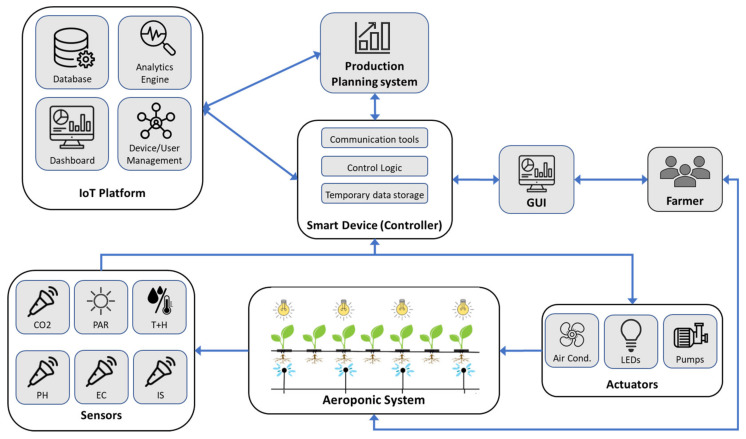
Context diagram of aeroponic system.

The use case is about soilless farming in controlled agriculture environments (referred to as vertical farming) that are characterized by a high degree of technology usage. Lettuce crops are grown in a completely controlled environment where all parameters relevant to plant growth are artificially provided, such as growth medium, irrigation, nutrition, and climatic conditions. The plants are grown in a growth chamber that consists of a number of sensors and actuators that are connected to a controller; each growth chamber with its associated sensors, actuators, and controller is referred to as a module. Each module sends its data to a remote database for storage and further advanced analytics. Also, a production planning system is connected to the IoT system via a specific API to acquire plant data. The overall IoT system is shown in the context diagram in Figure 8.

A monitoring digital twin is developed at the plant level (crop digital twin) with the objective of optimizing the growth of the lettuce plants. By considering the module as a production system that has inputs (seeds, energy, water, etc.) which are processed into outputs (plants, O_2_, inedible biomass), each plant stays in production from germination until maturity.

### 4.2. Aeroponic System Domain Model

**Figure 9:**
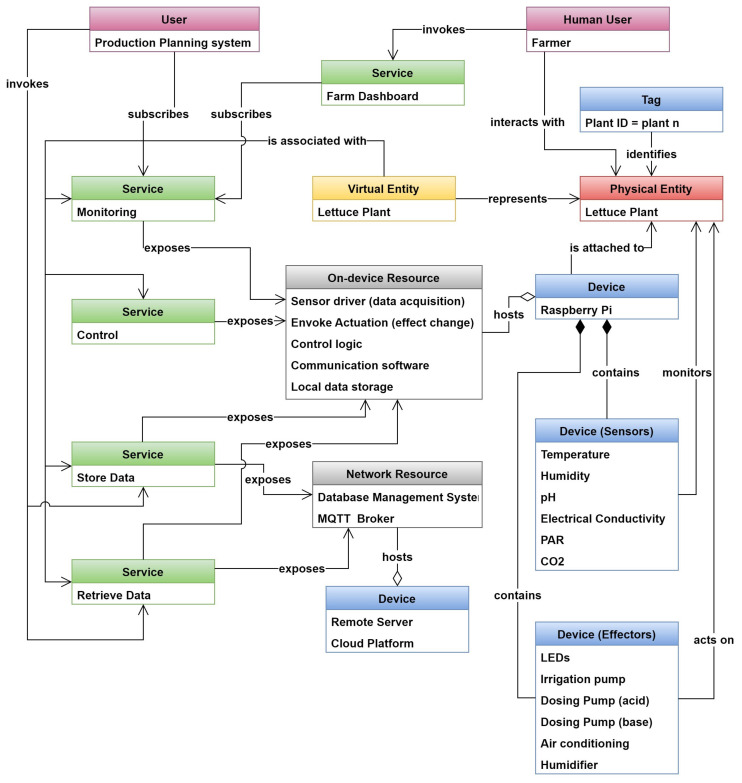
IoT domain model for lettuce plant in the aeroponics system.

As mentioned in the previous section, the IoT domain model serves as the foundation for the other sub-models by establishing a shared taxonomy for the primary concepts of IoT and their interconnections. This taxonomy forms the fundamental structure of the information model for a particular (IoT) domain. By using the modeling tools described in the previous sections, a domain model is created for the vertical farm system, as shown in Figure 9. The starting point is the physical entity under study, which is a single lettuce plant. This PE is identified by a specific tag that contains the plant ID. The plant is monitored by a set of sensors that collect information about the plant environment; these sensors feed their information to a Raspberry Pi [75]. The Raspberry Pi also controls various effectors (actuators) that act on the physical entity and its environment to provide the most suitable growth conditions. The physical entity is represented by a virtual entity and together they constitute the digital twin.

The virtual entity is associated with a set of services that either provide or acquire data to or from the virtual entity. In our model, we consider four types of services that are available to the virtual entity: The first is a monitoring service that acquires sensor data and updates the VE accordingly; the second is the control service that invokes a change in the physical environment by triggering an effector. A data storage service is used to store data in any data archive, whether it is a simple CSV file or a complex database; and finally, the data retrieval service acquires data from any data storage and uses it to update the VE.

All the above-mentioned services expose a set of resources; these resources can be on-device resources, i.e., resources hosted by the Raspberry Pi. These are sensor drivers used for data acquisition, control logic to trigger actuators, communication tools, and local data storage. It is noteworthy to mention that many other resources exist on the device (e.g., device operating system, GUI enabling software, etc.); however, our focus is on the high-level resources that interact directly with the VE services. Network Resources, on the other hand, are hosted on cloud or on some remote server with higher computational capabilities; these are the database management systems (DBMSs) that include the actual DB and the MQTT broker, which is responsible for IoT-related communications.

Two types of users are defined in this system: the first is a human who can be the farmer or a vertical farm operator; this user invokes the farm dashboard service, which in turn invokes the monitoring service to monitor the plant during its growth. This user also interacts directly with the PE for standard farm operations such as weeding, cleaning, and harvest, to name a few. The second user is the production planning system, responsible for managing plant production in the farm. This user subscribes to the monitoring service if it needs updated plant data, and it invokes the data storage and retrieval services in case it needs to acquire historical data or if it needs to update the DB.

### 4.3. Information View

The information model defines the structure, characteristics, and relationships of information managed by the virtual entity; it also defines the IoT services linked to the specific virtual entity. Based on the defined information model, we develop the information view that is specific to this use case.

**Figure 10:**
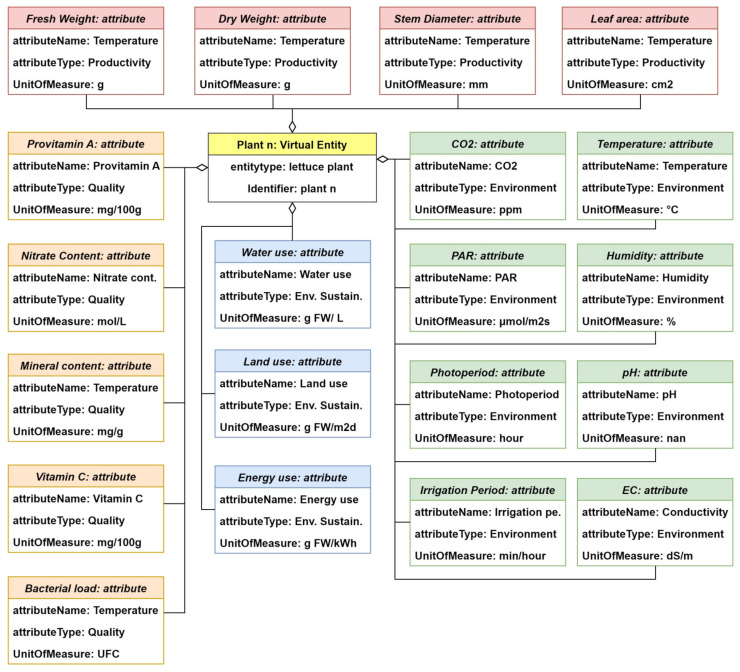
Information structure showing the virtual entity attributes (i.e., digital twin attributes).

Figure 10 shows the information structure of the digital twin. Starting from a digital twin of a single plant represented by a virtual entity “Plant n” that contains many attributes, these attributes hold the relevant information pertaining to the specific twin, as mentioned in the previous section. Information is provided or taken from the digital twin via services and the connection between the service and virtual entity is achieved through an association that maps the information of the service to a specific attribute. The type of information stored in the attribute can refer to the specific conditions of the physical entity such as the attribute “temperature”, or they can refer to KPIs that provide qualitative indicators about the physical entity, such as the indicator “Leaf area”. To this end, and by adopting the KPI formalization for aeroponic systems suggested by [76], the digital twin attributes are grouped as follows:

1. Environment attributes that provide information about the plant growth conditions (i.e., about the environment of the physical entity);
2. Productivity attributes which are KPIs that provide information about the overall biomass;
3. Quality attributes, which refer to vegetable quality;
4. Environmental sustainability attributes, that take into consideration the sustainability of the growth process.

The specific attributes associated with the VE are shown in Figure 10; each attribute contains the fields: attributeName, attributeType, which indicates the type of attribute in relation to the above-mentioned groups, and the unit of measurement. The productivity attributes Fresh weight, Dry weight, Stem diameter, and Leaf area are shown in red. Environmental attributes are in green, Quality in orange, and Environmental Sustainability in blue. Each of these attributes has a value container, which has a value and the metadata associated with it as defined by the IoT information model in Section 3. For the sake of simplicity, we will only describe the metainformation structure for a single attribute.

**Figure 11:**
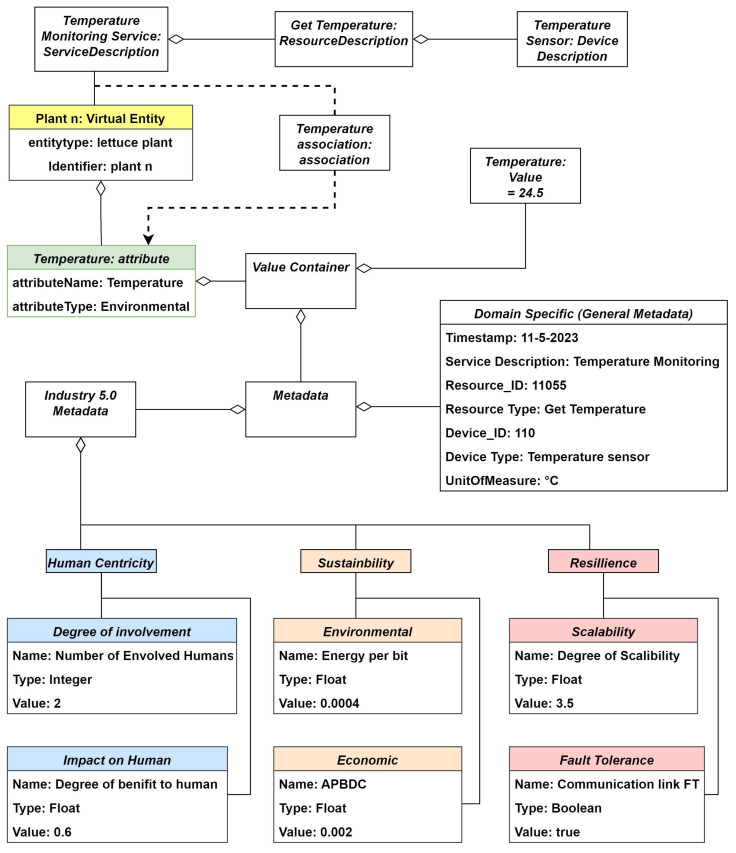
Information model for attribute “Temperature”.

Considering the attribute “temperature”, as shown in Figure 11, it has a value container categorizing one value and the zero-to-many information of the related value via means of metadata. The metadata are categorized into general metadata and Industry 5.0-specific metadata; the former contains fields such as the time stamp, the device issuing the data, and the resource used to obtain this information along with the service that delivered the information. Industry 5.0 metadata contains information about the sustainability, human-centricity, and resilience of the delivered information. In this example, we consider two metrics for each of these pillars. Namely, in terms of human-centricity, we observe the degree of human involvement and the impact of the delivered information on the human, for sustainability we use the metric average per-bit delivery cost (APBDC) to quantify economic sustainability and energy per bit for environmental sustainability. We do not consider social sustainability since it considers the social aspects outside of the system border and this is difficult to quantify within the scope of the work. For resilience, indicators of scalability and fault tolerance are used.

The information flow is initiated by a temperature sensor that sends the temperature value measured at the physical entity (the plant). This value is delivered to the attribute of the virtual entity associated with the temperature measurement through an association. This way, the measured value replaces the value in the temperature attribute of the virtual entity.

### 4.4. IoT System Architecture

**Figure 12:**
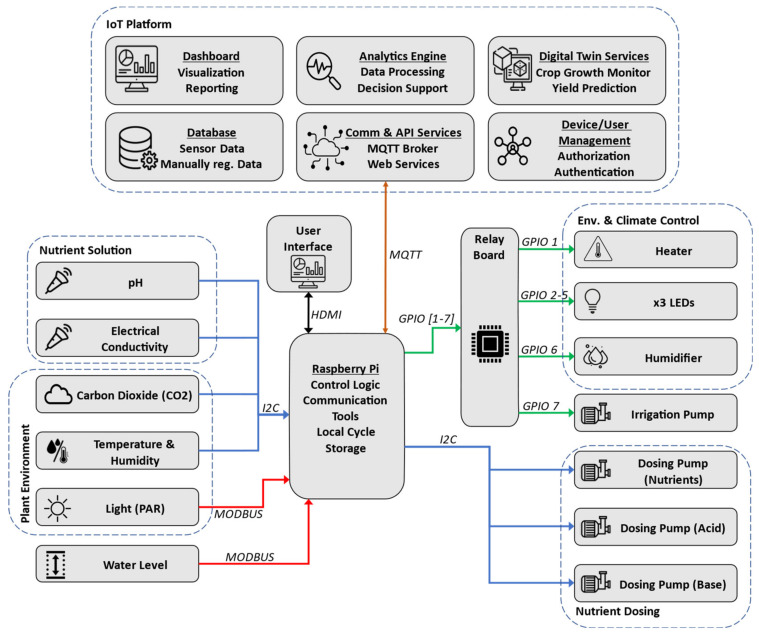
System architecture of developed aeroponics system.

Taking into consideration the elements described in the domain model and the information structure highlighted in the information view in the previous sections, the system architecture of the implemented aeroponic system is developed as shown in Figure 12; it consists of various sensors and effectors connected to a Raspberry Pi along with an IoT platform. The left portion of the diagram displays the sensors that function in two primary areas: the nutrient solution area, which is home to the pH, EC, and water level sensors; and the ambient environment of the plants, which includes the PAR, CO_2_, and temperature and humidity sensors.

The PAR and water level sensors transmit their data to the Raspberry using the Modbus protocol [77]. Modbus is an application layer communication protocol that has been considered the de facto standard for industrial automation for many years. Moreover, Modbus allows for a high degree of interoperability because it is an open standard. It is also a very cost-effective solution since the protocol can be supported by low-cost hardware due to its low complexity.

All the other sensors employ the I2C protocol to send their data. I2C, or Inter-Integrated Circuit, is a widely used serial communication protocol that facilitates communication between electronic devices [78]. With a straightforward two-wire design (SDA and SCL), I2C simplifies hardware implementation and reduces the pin count. Each device connected to the I2C bus has a unique address, allowing for the seamless integration of multiple components without conflicts.

The Raspberry Pi is considered the central hardware component in the system, as it is responsible for data acquisition, managing communications, control logic, and driving actuators depending on the outcome of the control logic. The main software component running in the Raspberry is Mycodo, which is an open-source environmental monitoring and automation system designed for home and industrial applications [79]. It is commonly used for tasks such as managing and controlling greenhouse environments, aquariums, and other settings where monitoring and automation are crucial. Moreover, Mycodo provides the tools for dashboard configuration and as a result, a graphical user interface is attached to the Raspberry Pi. The GUI shows sensor readings, trends, historical information, and notifications associated with the system.

Effectors (which include actuators and LEDs) are found on the right side of the diagram, similar to the sensors the effectors operate in the nutrient solution and the plant’s ambient environment. A heater is used alongside a humidifier to regulate the growth climate. A heater has been used in place of an air conditioner due to the cold climate where the chamber is implemented. Three LEDs are used to provide artificial night and day cycles. All these actuators are connected to the Raspberry Pi through its GPIO pins since they are controlled by standard ON/OFF switching. A relay board is used to allow for logic switching between the Raspberry Pi (3.3 V) and the power supply of the actuator (e.g., 220 V). Three dosing pumps are connected to the system via I2c; specifically, two dosing pumps are used for pH regulation (acid and base) and one for EC regulation.

The communication between the aeroponic system and the IoT platform is implemented using the MQTT protocol [80]. MQTT, which stands for Message Queuing Telemetry Transport, is a lightweight and open messaging protocol designed for low-bandwidth, high-latency, or unreliable networks. It is commonly used in the Internet of Things (IoT) to facilitate communication between devices. MQTT operates on the publish/subscribe model, where devices can publish messages to specific topics, and other devices can subscribe to those topics to receive the messages.

The IoT platform consists of six main modules. The first module is the communication and API module responsible for providing communication functionality to and from the platform; it contains tools such as the MQTT broker and the tools used for web services to provide machine to machine interaction across the network. These data are stored in the database module, which is a robust storage system that efficiently manages time series sensor data and user input data, ensuring data integrity and accessibility for analytics and reporting purposes. The analytics engine employs machine learning algorithms to process sensor data, offering descriptive, diagnostic, predictive, and prescriptive analytics for actionable insights and optimized decision support. The dashboard module offers a user-friendly interface providing real-time insights and historical trends, allowing stakeholders to visualize and analyze data generated by IoT devices, enhancing decision-making processes. Digital twin services enabling the creation of virtual representations of physical assets are provided by the DT module, facilitating growth monitoring and accurate yield predictions through continuous synchronization with real-world data. Finally, is the component used for device and user management for authorization and authentication; the function of this module is to provide secure access through strong authentication methods, role-based authorization, and the efficient administration of IoT devices and user accounts.

The developed architecture tries to take into account several qualities and requirements that are essential to IoT systems. First, in terms of interoperability, the realm of IoT contains many types of devices with different technologies and varying communication protocols. Several tactics such as building variation points in software and employing open-source tools and protocols can be used to help in this issue; to this end, Raspberry Pi, MQTT, Modbus, and Mycodo are used due to their open-source nature.

With regard to scalability, numerous strategies used in standard information systems such as trying to predict future system expansions, modular architecture design, and using scalable communication protocols can be used. However, compared to traditional information systems, it is even harder to cope with in a highly distributed and fast-evolving scenario, as we have in IoT. In the developed architecture, a Raspberry has been used due to the availability of I/O expansion boards (I/O shields); moreover, Modbus and I2C both allow for a maximum number of 255 on its communication bus, which is quite satisfactory for a single aeroponic module.

## 5. Conclusions

### 5.1. Summary of Scientific Contributions

The article introduces an IoT-DT framework, building upon the IoT-A reference architecture, to address the evolving requirements of Industry 5.0. This framework aims to describe IoT systems mainly focused on digital twin entities and capable of taking into account human-centricity, sustainability, and resilience. It recognizes and addresses the intricate interactions between virtual (digital twins) and physical entities, a core concept in digital twin technology. A pointwise description of the contributions of the research work presented by this manuscript is presented below.

- The proposed framework is built by focusing the IoT-A European reference architecture on the digital twin information holistic management (providing an integrated perspective on IoT systems and recognizing and addressing intricate interactions between virtual and physical entities) and by adding specific attributes and consideration regarding the increasingly popular European concept of Industry 5.0.
- The literature review section includes a systematic review of the the IoT-A concept to highlight existing research contributions and to synthesize scientific insights regarding the IoT-ARM project. The same section includes the state of the art regarding the technologies of digital twin and IoT systems appropriate in the 4.0 context and any research considering these technologies from a 5.0 perspective (scientific manuscripts regarding digital twin and human-centrality are mainly cited).
- The proposed framework provides a domain model that is robust within different domains of application (the new generation of smart farming and agriculture or Industry 5.0 environment for smart products and smart factories).
- The proposed functional model provides a further 5.0 characterization of the framework given by the management level of the model, i.e., the level that deals with types of decisions that are the most strategic and in common between different application areas.
- In the section dedicated to the case study, the information view referring to the information model for the vertical farming application is described through the textual contents of the subsection and Figure 11 regarding the information model for the attribute “Temperature”. The case study is a concrete guide for applications in a smart farming scenario, specifically, aeroponic systems in vertical farming, with the general objective of optimizing growth conditions through monitoring and control services, and it demonstrates versatility by engaging various stakeholders like human users, production resources, and other various stakeholders, referring to dedicated digital components.

### 5.2. Future Improvements

Looking ahead, we envision further enhancements and future directions that will fortify our framework’s utility.

- One critical area of exploration involves refining and expanding the metrics for Industry 5.0 requirements, especially for aspects like social sustainability and resilience.
- Real-world validation across diverse IoT scenarios is a natural progression to strengthen the practicality of our framework and, for this reason, works in progress are aimed at applying this methodology to three use cases in manufacturing, agricultural, and service sectors, to validate a general framework and to highlight common factors and specificities.
- Dynamic adaptation is another avenue we are eager to explore, delving into methods that allow our digital twin framework to dynamically respond to evolving Industry 5.0 standards and emerging technologies, especially for ensuring the resilience of the system.
- Additionally, future improvements will include an intensified focus on security and privacy aspects within the framework to ensure the robust protection of user data and interactions, aligning with the ever-growing importance of secure and private IoT systems.

## Data Availability

The data presented in this study are available in the manuscript text.
